# Tunable thermo-reversible bicontinuous nanoparticle gel driven by the binary solvent segregation

**DOI:** 10.1038/s41467-020-20701-3

**Published:** 2021-02-10

**Authors:** Yuyin Xi, Ronald S. Lankone, Li-Piin Sung, Yun Liu

**Affiliations:** 1 Center for Neutron Research, National Institute of Standards and Technology, Gaithersburg, MD 20899 USA; 2 Department of Chemical & Biomolecular Engineering, University of Delaware, Newark, DE 19716 USA; 3 Engineering Laboratory, National Institute of Standards and Technology, Gaithersburg, MD 20899 USA; 4 Department of Physics & Astronomy, University of Delaware, Newark, DE 19716 USA

**Keywords:** Soft materials, Colloids, Gels and hydrogels, Self-assembly

## Abstract

Bicontinuous porous structures through colloidal assembly realized by non-equilibrium process is crucial to various applications, including water treatment, catalysis and energy storage. However, as non-equilibrium structures are process-dependent, it is very challenging to simultaneously achieve reversibility, reproducibility, scalability, and tunability over material structures and properties. Here, a novel solvent segregation driven gel (SeedGel) is proposed and demonstrated to arrest bicontinuous structures with excellent thermal structural reversibility and reproducibility, tunable domain size, adjustable gel transition temperature, and amazing optical properties. It is achieved by trapping nanoparticles into one of the solvent domains upon the phase separation of the binary solvent. Due to the universality of the solvent driven particle phase separation, SeedGel is thus potentially a generic method for a wide range of colloidal systems.

## Abstract (web summary)

Bicontinuous porous materials made by colloidal self-assemblies have many applications. Xi et al. utilize colloidal particles dispersed in a binary solvent to form thermo-reversible bicontinuous gel structures with good reproducibility and scalability, and tunable structural and optical properties.

## Introduction

Colloidal self-assembly can form fascinating structures through either equilibrium or non-equilibrium routes. Gel formation, a non-equilibrium approach, shows tremendous usefulness to form novel structures not achievable with traditional equilibrium approach^1–4^. In particular, the formation of bicontinuous structures through gelation processes has attracted extensive research interests due to their important applications in energy storage^5^, membrane science^6^, flow-through reaction^7^, and cell delivery^8^. One of the widely studied bicontinuous structure systems is the bicontinuous interfacially jammed emulsion gel (Bijel), which can form porous materials with tortuous channels by arresting the solvent spinodal decomposition with colloidal particles trapped at the interface of two phase-separating solvents^2,9–12^. However, process-dependent structures formed through non-equilibrium routes make it very challenging to simultaneously optimize them to achieve reversibility, reproducibility, scalability, and tunability of formed structures. For example, for Bijel systems, the required fast quenching rate (from a few to tens of degrees per minute) has been restricting its scalability and thermal reproducibility^2,13^. Extensive works have been focused on simplifying the experimental requirements of Bijel systems, improving their large-scale processability, boosting the versatility and enhancing the control over domain size^4,9–11,14,15^.

**Fig. 1:**
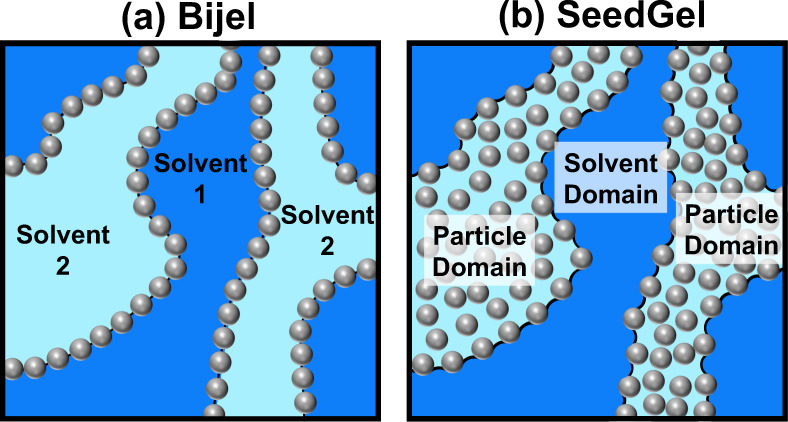
Schematic illustration of the differences between Bijel and SeedGel. **a** Bijel utilizes particles that wet both solvents equally (different solvent regions are defined by colors), whereas **b** SeedGel relies on favorable wetting of particles to one component of a binary solvent. Due to this surface wettability difference, particles in Bijel are jammed at the interface and SeedGel confines particles within one of the solvent regions.

To overcome the aforementioned challenges, a different type of colloidal gel that can also form bicontinuous structures is proposed and termed as solvent segregation driven gel (SeedGel). Similar to Bijel, colloidal particles dispersed in a binary solvent are used. However, different from Bijel, particles in a SeedGel are jammed within one solvent domain due to preferential wetting of particles by one component of the binary solvent upon solvent phase separation (illustrated in Fig. 1). Note that realizing bicontinuous structures with particles constraint within one of the liquid domains has been previously proposed in polymer blends and bicontinuous intraphase jammed emulsion gels (bipjel)^16,17^. However, there are major distinctions between SeedGel and the previous demonstrations. Whereas a strong attractive force between particles is needed for previous systems^16,17^, the particles in a SeedGel have a net repulsion or no attraction between them in liquid states. In addition, the SeedGel formation is driven by the local phase separation of solvents while the bulk solvent phase separation is required in the intraphase gelation of the previous systems^16,17^. These distinctions render SeedGel many unique and interesting properties. The structural arrest of particles in one solvent domain of SeedGel leads to the gel formation with great reversibility, reproducibility, scalability, and tunability. Compared to Bijel, SeedGel provides an alternative way to arrest bicontinuous structures by removing the often nontrivial surface treatment of particles, reducing the rigid requirement of fast quenching rate by orders of magnitude, allowing adjustable gelation temperature and domain size, and can easily incorporate particles as small as 10 nm. In addition, the use of the binary solvent offers SeedGel a unique opportunity to tune the optical property that is not achievable by many other gel systems.

## Results and discussion

**Fig. 2:**
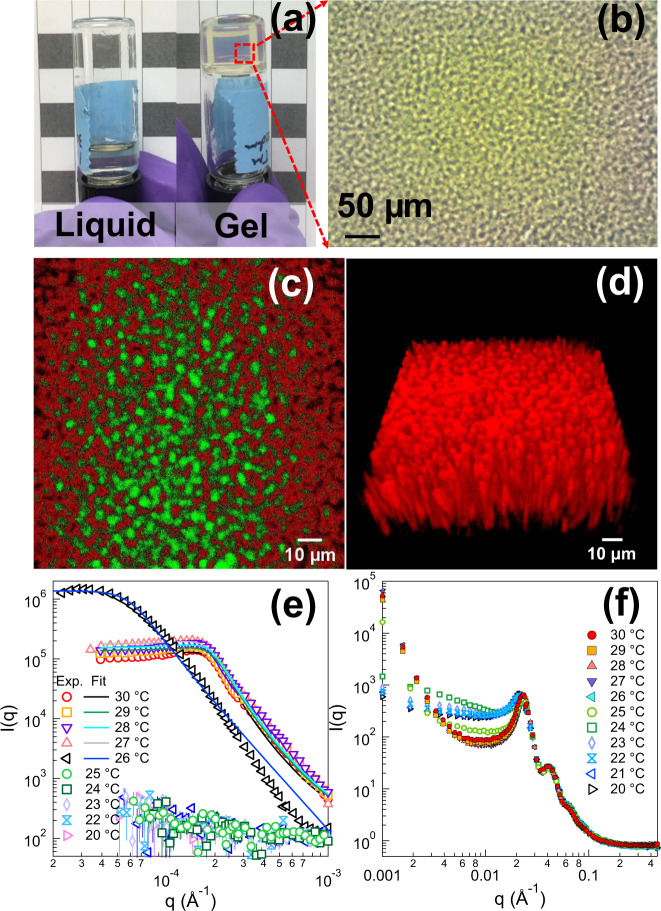
Properties and structures of a SeedGel sample characterized by different techniques. **a** Photographs of the sample at the liquid and gel (optically transparent) state. **b** Optical microscopic image of the gel state shows tortuous structures with the micrometer size. **c** A single plane (0.5-μm thick) and **d** 3-D confocal image with a total z-stack of 40 μm. Each plane of **d** shares the same x–y dimension as that of **c**. Note that the scale bar in **d** is based on the size in the x–y plane. **e** USANS and **f** SANS patterns of the gel structure at different temperatures by cooling a sample from a gel state (30 °C) to a liquid state (20 °C). By fitting the USANS patterns with the Teubner–Strey model, the averaged domain size can be obtained. The error bars in the figures represent one standard deviation and are often smaller than the symbol size.

To demonstrate the feasibility of forming a SeedGel shown in Fig. 1(b), spherical silica nanoparticles (diameter ~27 nm) dispersed in a mixture of water and 2,6-lutidine are used as a model system. The nanoparticle has high surface charge with its surface preferentially wetted by water^18^. As a result, the particle has a net repulsion between them. The solvent ratio is adjusted close to critical concentration of the bulk solvent (mass fraction of 28.4% 2,6-lutidine and 71.6% water in the solvent) with the particle volume fraction at 24.3%. This sample is in the liquid state at room temperature (20 °C) and forms gel at higher temperatures as shown in Fig. 2a.

A microscope image of the gel sample close to 29 °C (Fig. 2b) demonstrates a tortuous bicontinuous structure with a domain size on the order of a few micrometers. The bicontinuous domains are also visible in a single plane confocal microscope image and a 3-D image in Fig. 2c and d. Quantitative temperature-dependent characterizations of the multi-length scale structures are conducted with ultra-small angle neutron scattering (USANS) (Fig. 2e) and small angle neutron scattering (SANS) (Fig. 2f). These scattering patterns are dominated by silica particles since the scattering contrast between silica particles and the solvent is much larger than that between water and 2,6-lutidine. By analyzing the temperature-dependent scattering patterns, particle structures and gel transition temperature can be determined.

The gel state (above 26 °C) is indicated by a scattering peak at ~1.7 × 10^−4^ Å^−1^ in USANS patterns (Fig. 2e) due to the repeating distance between the tortuous particle domains. Reasonably good fit is achieved with the Teubner–Strey model that is widely used to describe the scattering pattern of a bicontinuous structure^19–21^. (See Supplementary Note 1 and Supplementary Table 1 in the Supporting Information for details.) A particle domain size of about 2 μm is obtained for this sample with the solvent channel size at around 1 μm at 30 °C consistent with the result of the microscope image shown in Fig. 2b–d. The solvent channel size can even reach submicrometer dimensions (The control of the domain size is discussed later in the main text in details.), which is comparable to the smallest achievable domain size of Bijel^4,10,13^. The gel sample consists of two tortuous domains: the particle domain that contains concentrated particles, and the solvent domain that is made of a mixture of water and 2,6-lutidine (Fig. 1b). The bicontinuous structure of the gel sample does not change between 30 °C and 27 °C as USANS peak position does not shift in this temperature range.

The gelation temperature of this particular sample is observed to be around 26 °C, which is 8 °C lower than the phase-separation temperature of the bulk solvent. This is expected as the gel transition is initiated by the attraction between particles due to the local phase segregation of the binary solvent^22–24^. (The mechanism is discussed later in the main text in details.) Around the gel transition temperature, the particle domain size increases to ~7 μm due to the weakened strength of gel resulting in the coarsening of domains^25^. Further cooling down the sample wipes out the scattering peak at low-q region, resulting in the transition from gel to liquid. In complement to USANS results, SANS patterns (Fig. 2f) are obtained to investigate the local structures of assembled particles upon cooling. In comparison to a broad interparticle structure factor peak in the liquid state, a much sharper peak at slightly larger q-position appears in the gel state, indicating that particles are packed much denser in a gel state with smaller average interparticle distance. The particle local volume fraction in the particle domain is about 39% in the gel state by fitting the SANS patterns^26,27^. (See Supplementary Note 2 and Supplementary Fig. 1 in the Supporting Information for details.) A wide range of temperature ramping rates are used to form gel even as slow as 0.1 °C/min shown in Supplementary Fig. 2 and Supplementary Note 3 of the Supporting Information, which is in sharp contrast to the fast quenching required by many Bijel systems^2,13^.

**Fig. 3:**
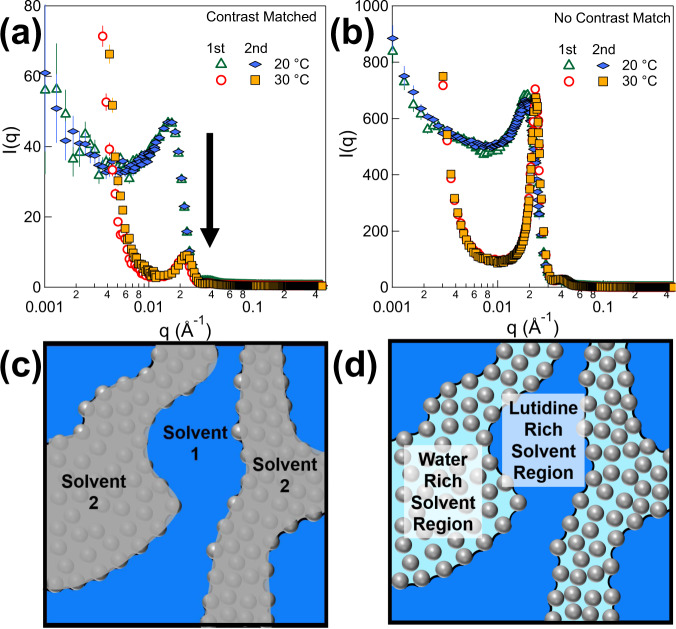
Demonstration of water rich solvent composition in the particle domain using contrast matched SANS experiments. SANS profiles of thermal reversible SeedGel prepared with 27 nm particles cycling between liquid state (20 °C) and gel state (30 °C). **a** Sample A implements a specially designed mixture of H_2_O and D_2_O to contrast match the scattering length density of water phase to that of silica. **b** Sample B uses normal water (H_2_O) so that silica particles are still visible to neutrons when engulfed by water. Schematic drawings illustrate **c** the case of water SLD matched to that of silica in sample A and **d** not matched condition in sample B. **c** and **d** correspond to the experimental conditions in **a** and **b**, respectively. The error bars in the figures represent one standard deviation and are often smaller than the symbol size.

In the gel state, the binary solvent also separates into two solvent regions commensurate with the observed bicontinuous structure: the water rich solvent region overlapping with the particle domain, and the lutidine rich solvent region coinciding with the solvent domain. Specially designed SANS experiments are used to probe the solvent separation. Two samples are prepared with the same particle and lutidine concentration, which are named as sample A and sample B. In the sample A, the scattering length density (SLD) of water is tuned to match that of particles (mixing H_2_O and D_2_O with the volume ratio at 48:52), while the sample B is prepared with normal water (H_2_O) as a reference. Their SANS results at different temperatures are shown in Fig. 3a and b, respectively. In the liquid state (20 °C), both samples show a peak at q = 0.016 Å^−1^ due to the interparticle structure factor. However, in the gel state, the peak intensity for the sample A shows a sharp intensity drop by more than five times while that of sample B remains about the same as that in a liquid state. The difference of the relative change of the intensity in a gel state observed in Fig. 3a and b is solely due to the solvent composition difference. For the sample A, the scattering intensity drops since the solvent surrounding particles is rich in matched water. And there is no contrast between the matched water and particle in a gel state, i.e., silica particles become almost invisible as illustrated in Fig. 3c. In comparison, particles are visible in both liquid and gel states for the sample B prepared with normal water (H_2_O) illustrated in Fig. 3d. The results thus indicate that the particles in the gel state are fully engulfed by the water rich solvent. The scattering peak does not completely disappear in Fig. 3a as the water rich solvent region still has small amount of lutidine as expected for a binary solvent phase separation. The relative volume fraction in the water rich solvent region is estimated to be ~90% of water and 10% lutidine with a significant decrease of lutidine concentration from the nominal concentration of 30% volume fraction. (See Supplementary Note 4 in the Supporting Information for details). The formed gel is thus indeed a SeedGel with particle and solvent partition schematically illustrated in Fig. 1.

The formed gel has excellent thermo-reversibility, precise structural reproducibility, stability, and tunability. The same samples in Fig. 3a and b are measured twice by cycling the temperature between 20 °C and 30 °C. The SANS scattering patterns together with the USANS results shown in Supplementary Fig. 3(a) in Supplementary Note 5 of the Supporting Information are completely identical at the same temperatures in gel states. Thus, both the local and large domain structure are not only thermally reversible, but also precisely reproducible when following the same heating protocol. In addition, the gel structure is very stable after at least 20 h of aging at 30 °C and no change of domain structures is observed by USANS within a reasonable duration of period. (Details are in Supplementary Fig. 3(b) in Supplementary Note 5 in the Supporting Information.)

**Fig. 4:**
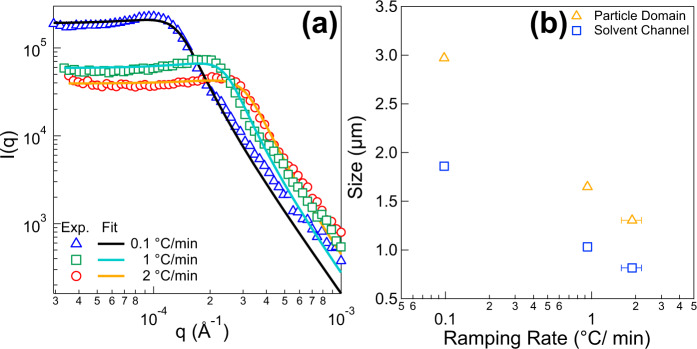
Domain size tunability of SeedGel by controlling the temperature ramping rate. **a** USANS profiles of SeedGel formed with 27 nm particles at 30 °C. Three different ramping rates are used to form SeedGel from 4 to 30 °C to show structure tunability. The temperature change rates from fast ramping to slow quenching are ~2 °C/min, 1 °C/min, and 0.1 °C/min. The domain size can be controlled by using different ramping rates and the scattering data is fit with Teubner–Strey model. **b** The obtained particle domain and solvent channel sizes are plotted as a function of ramping rates. Note the ramping rate here is the actual temperature change speed within the sample. It is carefully calibrated by recording the real time temperature change with a thermocouple placed inside the sample cell at each set ramping rate. The error bars in the figures represent one standard deviation and are often smaller than the symbol size.

Besides thermo-reversibility and stability, the domain size of SeedGel is tunable by controlling the temperature ramping rate. By comparing USANS profiles at three different ramping rates, the domain size is shown to be dynamically adjusted (Fig. 4 a). Faster ramping rate leads to a smaller domain size, whereas a slower quenching results in a larger domain size. By fitting the USANS data with the Teubner–Strey model, the fast ramping rate could push size of particle domain down to around 1.3 μm and solvent channel to about 800 nm, as shown in Fig. 4b. The slow ramping rate, on the other hand, increases the particle domain size to 3 μm and solvent domain width to 1.8 μm (Fig. 4b), which are almost three times the size of that produced with fast ramping rate. Thus, for a SeedGel, controlling the ramping rate is a simple and effective way to change the size of the bicontinuous channels due to its thermo-reversibility and stability (Fig. 3 and Supplementary Fig. 3 in the Supporting Information).

The SeedGel formation mechanism is different from that of Bijel. For Bijel, the bulk solvent phase separation of the binary solvent initiates the gel formation. In contrast, the local phase segregation of the binary solvent confined between particles initiates the gelation process for SeedGel, which in turn drives the macroscopic solvent phase separation commensurate to the particle domain. Supplementary Fig. 4 in the Supporting Information shows schematic pictures of our current understanding of the SeedGel formation (Supplementary Note 6). It is well known that an effective attraction between particles can be generated in a binary solvent when the solvent condition is moving towards its phase-separation point. This solvent introduced attraction has been widely termed as the Critical Casmir force^18,28,29^. Despite the current debate on the exact physical origin of this attraction^24,29,30^, the consensus of the community is that when the surface of particles is preferred to be wetted by one individual component in a binary solvent, it can develop an adsorption layer on particle surface. The thickness of this preferentially adsorbed solvent layer keeps growing when the binary solvent moves closer to the phase-separation temperature. In our case, ramping up the temperature increases the thickness of water rich adsorption layer on silica particle surface, which has been well studied in literature^18,23^. A recent experiment shows that at large particle concentrations, the attraction is driven by the local solvent segregation (or capillary condensation) of the binary solvent confined between neighboring particles (shown schematically in Supplementary Fig. 4(b))^23^. It generates a bridging attractive force to bring two neighboring particles close to each other. Both the wettability and thickness of the water layer on the particle surface play roles in this solvent introduced attractive force between particles. The surface chemistry determines the water concentration at the particle-liquid interface (i.e., wettability) and the correlation length of the solvent fluctuation dictates the thickness of the adsorption layer. These bonded particles can grow into larger clusters by incorporating more particles (Supplementary Fig. 4(c)). This is consistent with what is observed in our SANS patterns, in which a broad peak is developed at about 0.004 Å^−1^ right before the formation of the gel (Fig. 2f and Supplementary Fig. 2). The small particle size (around 27 nm in diameter) limits their interstitial space (or pore space) when particles are densely packed in clusters with pore size around 5 nm. The small interstitial space causes further solvent segregation to drive most lutidine out of clusters (See the schematic figure in Supplementary Fig. 4(c)). These particle clusters continue growing to form the particle domain, which percolate to form a gel with a bicontinuous structure (Supplementary Fig. 4(d)). It also results in a water rich solvent in the particle domain. This is supported by a recent experimental evidence that at the gelation transition temperature of our sample (26 °C), the adsorbed water layer on the surface of the same type of silica particle (27 nm in size) has a thickness around 6 nm that is comparable to the pore size formed by packed particles^23^. The majority of particles are within the particle domain in SeedGel. Only a small fraction of particles within the particle domain are near the domain interface. Note that the capillary condensation introduced attraction has been also found useful for other gel and granular systems too^31^. When the temperature is decreased, the two components of the binary solvent becomes miscible again. The solvent fluctuation induced attraction between particles becomes negligible. The net repulsion between charged silica particles makes them well dispersed in the liquid state. Therefore, to enable the reproducibility of the SeedGel, there should be no strong attraction between particles in liquid state. As the solvent segregation induced attraction between particles is a generic interaction commonly observed for particles dispersed in a binary solvent, SeedGel is thus potentially a general mechanism that can be applied to other functional particles or different binary solvents to achieve great tunability.

**Fig. 5:**
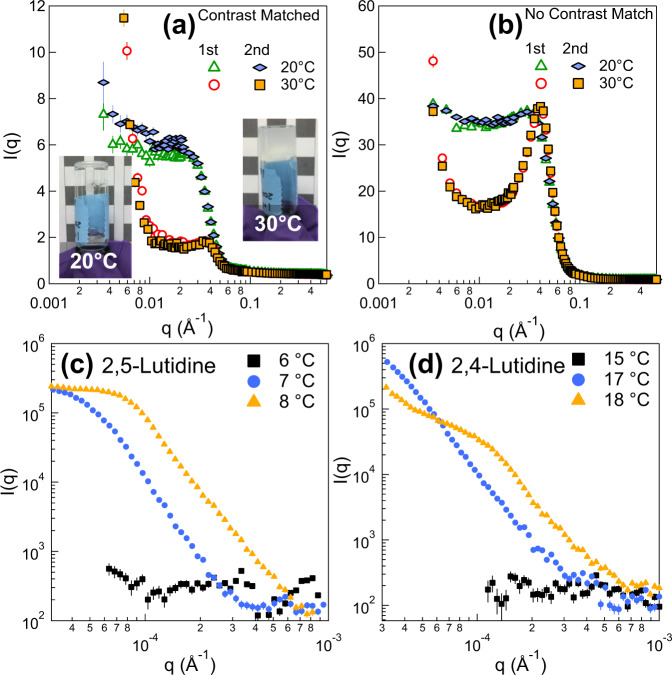
Thermal reversible SeedGel prepared with 10 nm particles and different binary solvents probed by SANS and USANS. The SLD of water phase is **a** matched to and **b** not matched to that of silica. Similar to what is demonstrated with 27-nm particles, the 10-nm particles are jammed in the water rich solvent region, which makes SeedGel possible for really small particles. Since the adsorption energy at the interface decays quadratically with the decrease of particle size, it is challenging to form Bijel with small nanoparticles. Owing to its sensitive and reversible temperature response, the ability to tune the gelation temperature of a SeedGel broadens its applications. The tunability of transition temperature can be simply adjusted with isomers of 2,6-lutidine in SeedGel. The USANS scattering profiles of 2,5-lutidine and 2,4-lutidine at varied temperatures are shown in **c** and **d**, respectively. The error bars in the figures represent one standard deviation and are often smaller than the symbol size.

To test the generality on different sizes, Fig. 5a and b demonstrate feasibility, thermal reversibility, and structural reproducibility of SeedGel using spherical silica particles with 10 nm diameter by performing similar experiments shown in Fig. 3a and b^32^. Note that the 2nd curve in Fig. 5a shows slightly higher intensity of low-q scattering when compared to that of the 1^st^ curve in the liquid state at 20 °C. This change is due to the fact that a small amount of random sized aggregates that are not fully dissolved in the sample after the previous formation of SeedGel due to its slow kinetics. The fast advancement in nanoscience has successfully produced many interesting colloidal particles with size down to sub-10-nm in the past decades. One of the most successful examples is quantum dot with unique electrical and optical properties. SeedGel works very well with small size particles, opening up a window for many interesting colloidal particles with size down to 10 nm that are rather difficult to form Bijels^4,9,10,13,33^.

SeedGel has a broad range of adjustable gelation temperature as the gelation temperature can be tuned by controlling the property of the binary solvent. Same silica particles (27 nm diameter) used in Fig. 2 are dispersed in binary solvents by replacing 2,6-lutidine with 2,5-lutidine and 2,4-lutidine. The SeedGel formation is observed with the corresponding gel transition temperature at 7 and 17 °C, respectively (Fig. 5c and d) due to the fact that changing solvent isomers shifts the bulk phase temperature of a binary solvent. The lower scattering intensity of the sample prepared with 2,5-lutidine at the gel transition temperature (7 °C) is due to the fact that the formed domain size is too large, which is outside the q-range accessible by USANS. The tunability of gelation temperature greatly expands the applications of SeedGel by designing its structure and property response to external stimuli at will.

The optical properties of SeedGel can also be controlled by adjusting the refractive index between the particle domain and solvent domain. The refractive index of 2,6-lutidine (*n* = 1.497) and silica (*n* = 1.46) are both larger than that of water (*n* = 1.33). When increasing the temperature, the particle domain loses 2,6-lutidine while the solvent domain gains lutidine because 2,6-lutidine is less soluble in water at higher temperatures. Consequently, the refractive index of the particle domain decreases and that of the solvent domain increases without the change of particle domain structures. As a result, the sample in Supplementary Fig. 5 in Supplementary Note 7 in the Supporting Information is initially turbid at the gelation transition temperature (about 26 °C), but slowly becomes transparent at about 29 °C, similar to the sample shown in Fig. 2(a). This thermally reversible transparent SeedGel can also be obtained at varied particle concentrations (Supplementary Fig. 6 in Supplementary Note 7 in the Supporting Information). The finely adjustable optical property is thermo-reversibly driven by the solvent phase separation. Bijel samples and many other gel systems with micrometer sized domains are usually turbid. The formation of the transparent particle gel with micrometer sized bicontinuous domains introduces new opportunities for SeedGel to many light sensitive applications, such as sensors or transparent bicontinuous mold/micro-reactors for inducing photo-crosslinking of other materials.

In conclusion, SeedGel can be realized by tuning nanoparticle surface to be strongly favored by one component of a binary solvent and no net attraction exists between particles in the liquid state. It shows excellent thermal reversibility, precise structural reproducibility, and tunable domain sizes at micrometer length scales. SeedGel is extremely versatile and is demonstrated here to work with different particle sizes and have a large range of adjustable gelation temperatures. One amazing property of the SeedGel is its tunability of the refractive index of different domains that is tough to achieve by physical gel systems with micrometer sized domains. As majority of colloidal particles are aqueous based, SeedGel provides an alternative route to achieve bicontinuous structures for many water-soluble particles that do not form Bijel. Thus, SeedGel complements Bijel systems and greatly extends the applications of bicontinuous structures formed by colloidal self-assembly. As the gel formation is initiated by the local binary solvent phase separation, it is expected that this is a general approach that has potential to work with many different types of particles. Future works will be needed to evaluate its applications with other type of particles and binary solvents.

## Methods

### Materials

Chemicals and solvents were used as received without further purification. 2,6-lutidine (anhydrous, 99%), 2,4-lutidine (99%), 2,5-lutidine (95%), Ludox SM (mass fraction of 30%), Ludox TM-50 (mass fraction of 50%), and Rhodamine B (95%) were purchased from Sigma–Aldrich (St Louis, MO). Deuterium oxide (D, 99.9%) was obtained from Cambridge Isotopes (Tewksbury, MA).

### Preparation of SeedGel

Ludox silica particles (Ludox TM (pH = 9) and Ludox SM (pH = 10)) are highly charged silica particles. The SeedGel sample was prepared by mixing silica dispersions in water with lutidine at a specific ratio. Mixing lutidine and water does not alter the pH value so that all silica particles remain highly charged. The average size of TM and SM particles is about 27 and 10 nm, respectively. To form SeedGel with 27-nm particles, lutidine volume of 300 μl is added to each milliliter (ml) of Ludox TM solution. It corresponds to a weight fraction of 28.4% 2,6-lutidine and 71.6% water in the solvent. Ludox SM is used to form gel with 10-nm particles. Lutidine volume of 200 μl is added to gel the sample for every ml of Ludox SM, which is equivalent to weight fraction of 17.8% 2,6-lutidine and 82.2% water in the solvent.

In this work, 300 and 200 ul 2,6-lutidine were used to generate SeedGel with 28 and 10 nm particles, respectively. The gelation temperature was shifted by mixing 300 μl 2,4-lutidine or 2,5-lutidine with 1 ml of Ludox TM solution. The solutions were vigorously vortexed after mixing at desired ratios, which may cause the formation of a temporary gel at room temperature. Subsequent waiting time of a couple of days would dissolve this temporary gel at room temperature, which led to a homogeneous solution that can form thermo-reversible gel at elevated temperatures. In the process of dissolving the initially formed gel, repeated vortex or continuous low shear applied by a roller were observed to be helpful during the first day of mixing. For SeedGel with 10 nm particles, a waiting time of about 20 h was used to prepare a sample after 2,6-lutidine was mixed with Ludox SM solution.

### Small angle neutron scattering (SANS) and ultra-small angle neutron scattering (USANS)

Both SANS and USANS experiments were conducted at NIST center for neutron research (NCNR, Gaithersburg, MD). Both NG7 and NGB30 were used to collect SANS results^34^. Scattering patterns from three detector positions, including lens, were recorded to provide a q-range of 0.001 Å^−1^ < q < 0.45 Å^−1^. Peltier-Driven heating/cooling blocks were utilized to control temperatures with a precision of ±0.1 °C. At least 30 min waiting time was implemented before measuring the scattering data after each temperature change to ensure that the equilibration was achieved. The sample was sealed by a demountable titanium cell with a path length of 1 mm with a Teflon coated O-ring. The scattering from a pair of quartz windows were properly subtracted. The open beam flux and transmission of each sample at every detector position was recorded to bring the scattering data to absolute intensity. The USANS experiments were conducted on BT-5 to cover a q-range between 3 × 10^−5^ Å^−1^ and 0.001 Å^−1^. The temperature was controlled by circulation thermal bath. All the data reduction was performed using standard Igor (Wavemetrics, Portland, OR) macros^35^. The fitting of the results was conducted using the SASView software^36^.

For the contrast matching experiment using SANS, D_2_O was mixed with Ludox TM/SM solution so that the volume ratio of D_2_O to H_2_O in the dispersion was 58:42. The scattering length density (SLD) of water phase was thus matched to that of silica. The addition of D_2_O only slightly changed the solution PH value (by about 0.3), which did not alter the stability of silica nanoparticles. An Amicon centrifugal filter unit (Ultra-15, MilliporeSigma, Burlington, MA) equipped with a membrane of 3 kDa pore size was used to filtrate out excess water. Centrifugation (Centrifuge 5702, Eppendorf, Hauppauge, NY) with relative centrifugal force (rcf) of 3000 × *g* for around 30 min was used to concentrate nanoparticles close to their original volume fraction. Then desired volume of 2,6-lutidine was added to contrast matched silica dispersion for SeedGel formation with 27 nm and 10 nm particles.

### Confocal microscopy

Laser scanning confocal microscopy (LSCM, Zeiss model LSM 800, Thornwood, NY) was performed in fluorescence mode with a 561 nm laser, ×50/0.5 air lens, an aperture size of 119 µm, and at a scanning z-step of 0.5 µm. A florescent dye (Rhodamine B) that preferentially partitions into the 2,6-lutidine rich phase is used. Fluorescence light emanating from the sample was collected from 576 to 700 nm. Imaging was performed on SeedGel at 28.4 °C. The temperature was controlled with a customized sample holder that tightly fit a quartz sample cell. A thermocouple was placed in the vicinity of the quartz cell to measure the temperature. External thermal bath was connected to the sample holder to maintain a constant temperature around the sample cell. Images were collected from a scanning area of 127.8 × 127.8 µm. Data collection was performed with Zen Blue (Zen 2.5 System) and image analysis was performed with Image J^37^.

## Supplementary information

Supplementary Information

## Data Availability

All data that support the finding of this work are available from the corresponding author upon reasonable request.
