# Supplementary material for: Tunable thermo-reversible bicontinuous nanoparticle gel driven by the binary solvent segregation
Source: Nat Commun. 2021 Feb 10;12:910. doi: 10.1038/s41467-020-20701-3 (PMC7876140; doi:10.1038/s41467-020-20701-3)
Supplement: Supplementary file 1 — Supplementary Information [file 41467_2020_20701_MOESM1_ESM.pdf]

Supporting Information

Tunable thermo-reversible bicontinuous nanoparticle gel driven by the binary solvent segregation

Yuyin Xi [1,2], Ronald S. Lankone [3], Li-Piin Sung [3], Yun Liu [1,2,4*]

1. Center for Neutron Research, National Institute of Standards and Technology, Gaithersburg, MD, 20899, USA
2. Department of Chemical & Biomolecular Engineering, University of Delaware, Newark, DE, 19716, USA
3. Engineering Laboratory, National Institute of Standards and Technology, Gaithersburg, MD, 20899, USA
4. Department of Physics & Astronomy, University of Delaware, Newark, DE, 19716, USA

Supplementary Note 1: The Teubner-Strey Model for the ultra-small angle neutron scattering (USANS) fitting to obtain the domain size information

Teubner-Strey model is widely used to fit the bicontinuous structure of a two-component system. [1–3] The scattering intensity can be modeled using the correlation length (ξ) and periodicity (d). [1–3] Their relationship is illustrated in Supplementary Equation 1 through Supplementary Equation 4.

$$I(q) = \frac{8\pi\varphi_a(1-\varphi_a)(\Delta\rho)^2 c_2/\xi}{a_2+c_1q^2+c_2q^4} \quad (1)$$

$$a_2 = [1 + (\frac{2\pi\xi}{d})^2]^2 \quad (2)$$

$$c_1 = -2\xi^2(\frac{2\pi\xi}{d})^2 + 2\xi^2 \quad (3)$$

$$c_2 = \xi^4$$

where $\Delta\rho$ is the difference of scattering length density (SLD) and φ_a is the volume fraction of one domain, which can be the volume fraction of either the particle domain or the solvent domain. The fitting was performed by fixing other parameters except the periodicity (d), correlation length (ξ), and scale. The reason to let scale vary is to account for the dynamic contrast change at varied temperatures between particle and solvent domains, which originates from the change of lutidine concentration in both water-rich and lutidine-rich solvent regions. The periodicity distance includes both the average size of the particle domain and solvent domain. If the ratio of the average domain size between the solvent and particle domain is approximately the volume ratio of these two domains, the average size of the particle domain is estimated to be about 2 µm, and the size of the solvent channel is about 1.3 µm at 30 ˚C. At 26 ˚C, the particle domain increases to 7.1 µm and the solvent channel to 4.5 µm.

Supplementary Table 1. The resulted periodicity and correlation length from Teubner-Strey model fitting of USANS data at different temperatures.

**Temperature (˚C)**	**Periodicity (µm)**	**Correlation Length (µm)**
30	3.34	1.80
29	3.38	1.73
28	3.37	1.75
27	3.52	1.80
26	11.6	3.66

Supplementary Note 2: Estimation of the particle volume fraction in the particle domain with small angle neutron scattering (SANS) fitting using the Hayter-Penfold method

In order to estimate the volume fraction of particles in the particle domain, SANS data at relatively high-q is fitted using the Hayter-Penfold model. Because the domain size is much larger than the particle diameter, most particles in the particle domain are not at the interface. As no additional salt is added into the solution and particles are highly charged, a strong long-range screened charge repulsion exists between particles.[4] Therefore, in order to calculate the inter-particle structure factor with a long-range repulsion, the Hayter-Penfold model is used. [5,6] We have attempted to fit the SANS pattern at high-q with other interaction models, such as pure hard sphere interaction, and the interaction with a short-range attraction and long-range repulsion. The charge interaction model gives the best fitting results.

To fit the scattering results of concentrated particle solutions, an independent experiment is performed to obtain the information of the form factor: the radius of the sphere and the size polydispersity by fitting a scattering curve of a dilute particle dispersion in water (volume fraction of 0.5 %). (The SANS pattern is shown together with the fitting in Supplementary Fig. 1(a).)

Supplementary Fig. 1(b) shows the SANS profile of a gel sample together with its fitting. The fitting only focuses on relatively high-q region as we are only interested in the particle arrangement in the particle domain. The model describes the results reasonably well. In this model, the scale factor, volume fraction of spheres, as well as the charge on the sphere surface are used as fitting parameters. All the rest of the parameters are fixed at constant values. The scale factor is determined to be 0.697 and the local volume fraction to be 0.391.

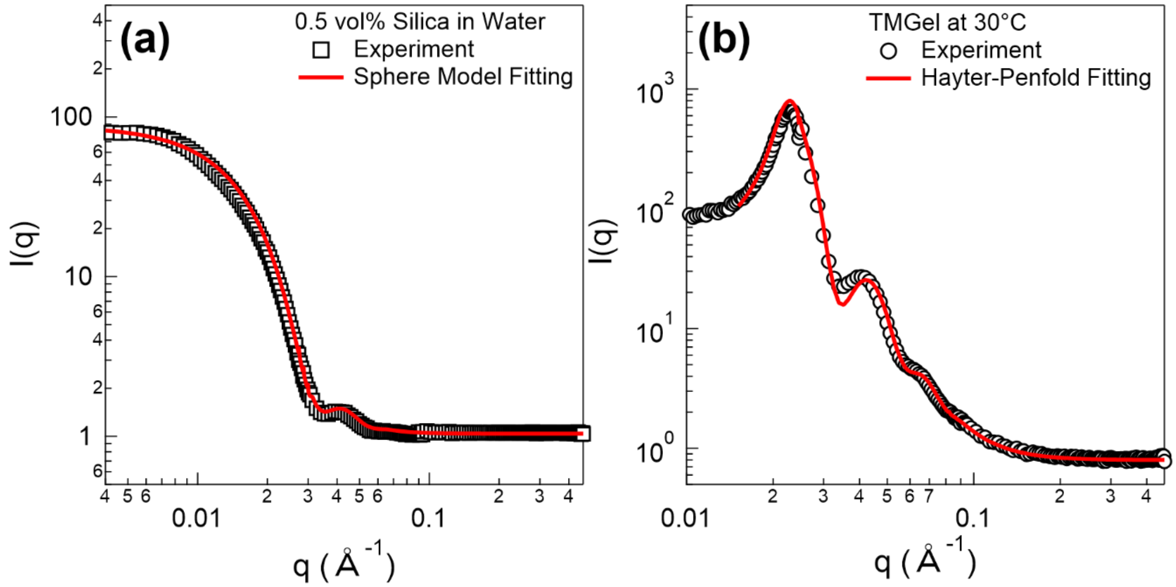

Supplementary Fig. 1. (a) Form factor fitting of small angle neutron scattering (SANS) profile with a sphere model for a dilute silica dispersion (volume fraction of 0.5 %) in water and (b) Fitting of the SANS profile at high-q range of SeedGel with 27 nm particles formed at 30 ˚C with Hayter-Penfold method.

Supplementary Note 3: Quasi-static heating leads to the SeedGel formation

We tested the structure of SeedGel by increasing the temperature from liquid to its gel state. SANS profiles are recorded at each degree upon heating. Considering the time for the measurement, ramping and equilibration, it takes about an hour at every temperature point. In fact, gels can successfully form even with a very slow ramping rate (0.1 ˚C/min) as discussed in Figure 4 in the main text. This is in sharp contrast to the fast quenching rate required in many Bijel systemes.[7] The quasi-static temperature change relaxes the restriction in sample size and makes the gel formation easily scalable to a large sample volume. At the same time, the ability to generate SeedGel even at very slow ramping rate provides an additional knob to control the domain size, which is discussed in Figure 4 in the main text.

Similar to Figure 2(c) in the main text, the charge repulsion dominates the interactions between silica particles at 20 ˚C. A well-defined peak at high-q (approximately 0.023 Å^{-1}) is due to the

correlation between nearest neighbor particles with a strong charge repulsion. Right below gelation transition temperature (24 ˚C), a broad peak at q-values between 0.001 Å^{-1} and 0.01 Å^{-1} is observed. This indicates that the attraction introduced by solvent fluctuation between particles is reasonably strong to cause the particle aggregation. Further increasing the temperature leads to the SeedGel formation with particle domains percolating throughout the whole sample volume. This is indicated by the Porod's law behavior with a q^{-4} decay at the low-q range.

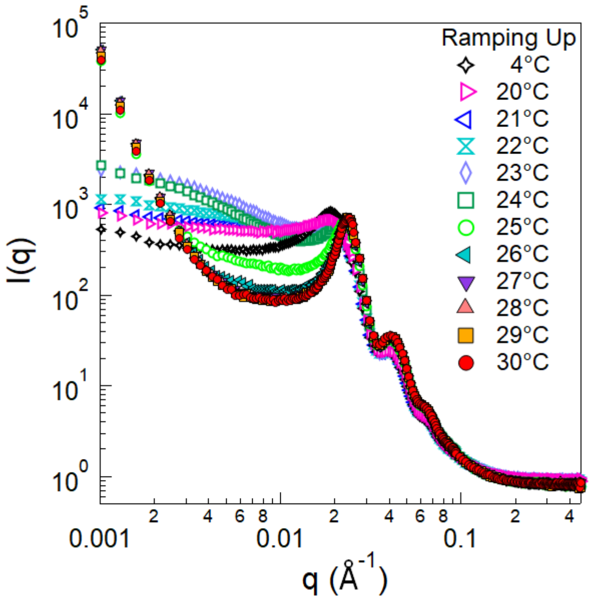

Supplementary Fig. 2. SANS profiles of SeedGel recorded at every degree upon heating from liquid state to gel state. The error bars in the figure represent one standard deviation.

Supplementary Note 4: Estimation of the relative concentration of lutidine and water in the particle domain

The 2,6-lutidine ratio in the particle domain is estimated based on the results from contrast matching experiment shown in Figure 3(a) and 3(b) in the main text. It is noted that the intensity of the structure factor peak reduces by more than five times when changing from the liquid state to the gel state for this sample. This is due to the enrichment of water concentration in the particle domain. By using the SANS intensities of the inter-particle structure factor peak at different temperatures, the concentration of lutidine can be calculated with the Supplementary Equation 5 through Supplementary Equation 15.

$$\frac{I(A,\ 20^{\circ}C)-I_{\mathrm{bkg}}}{I(A,\ 30^{\circ}C)-I_{\mathrm{bkg}}}=\frac{(\Delta\rho(A,\ 20^{\circ}C))^{2}P(20^{\circ}C)S(20^{\circ}C)}{(\Delta\rho(A,\ 30^{\circ}C))^{2}P(30^{\circ}C)S(30^{\circ}C)} \quad 5$$

$$\frac{I(B,\ 20^{\circ}C)-I_{\mathrm{bkg}'}}{I(B,\ 30^{\circ}C)-I_{\mathrm{bkg}'}}=\frac{(\Delta\rho(B,\ 20^{\circ}C))^{2}P(20^{\circ}C)S(20^{\circ}C)}{(\Delta\rho(B,\ 30^{\circ}C))^{2}P(30^{\circ}C)S(30^{\circ}C)} \quad 6$$

$$\frac{\left(\frac{I(A,\ 20^{\circ}C)-I_{\mathrm{bkg}}}{I(A,\ 30^{\circ}C)-I_{\mathrm{bkg}}}\right)}{\left(\frac{I(B,\ 20^{\circ}C)-I_{\mathrm{bkg}'}}{I(B,\ 30^{\circ}C)-I_{\mathrm{bkg}'}}\right)}=\frac{(\Delta\rho(A,\ 20^{\circ}C))^{2}(\Delta\rho(B,\ 30^{\circ}C))^{2}}{(\Delta\rho(A,\ 30^{\circ}C))^{2}(\Delta\rho(B,\ 20^{\circ}C))^{2}} \quad 7$$

$$\Delta\rho(A,\ 20^{\circ}C)=SLD_{\mathrm{silica}}-(\varphi_{\mathrm{A,Lutidine,t}}SLD_{\mathrm{Lutidine}}+\varphi_{\mathrm{matchedwater,t}}SLD_{\mathrm{matchedwater}}) \quad 8$$

$$\Delta\rho(A,\ 30^{\circ}C)=SLD_{\mathrm{silica}}-(\varphi_{\mathrm{A,Lutidine,d}}SLD_{\mathrm{Lutidine}}+\varphi_{\mathrm{matchedwater,d}}SLD_{\mathrm{matchedwater}}) \quad 9$$

$$\Delta\rho(B,\ 20^{\circ}C)=SLD_{\mathrm{silica}}-(\varphi_{\mathrm{B,Lutidine,t}}SLD_{\mathrm{Lutidine}}+\varphi_{\mathrm{H_2O,t}}SLD_{\mathrm{H_2O}}) \quad 10$$

$$\Delta\rho(B,\ 30^{\circ}C)=SLD_{\mathrm{silica}}-(\varphi_{\mathrm{B,Lutidine,d}}SLD_{\mathrm{Lutidine}}+\varphi_{\mathrm{H_2O,d}}SLD_{\mathrm{H_2O}}) \quad 11$$

$$\varphi_{\mathrm{A,Lutidine,t}}=1-\varphi_{\mathrm{matchedwater,t}} \quad 12$$

$$\varphi_{\mathrm{A,Lutidine,d}} = 1 - \varphi_{\mathrm{matchedwater,d}} \qquad 13$$

$$\varphi_{\mathrm{B,Lutidine,t}} = 1 - \varphi_{\mathrm{H_2O,t}} \qquad 14$$

$$\varphi_{\mathrm{B,Lutidine,d}} = 1 - \varphi_{\mathrm{H_2O,d}} \qquad 15$$

where A and B denote samples with and without SLD of water matched to that of silica, as described in the main text. *I*(20 ˚C) and *I*(30 ˚C) are the peak intensity of SANS pattern at 20 ˚C and 30 ˚C. I_{bkg} and $I_{\mathrm{bkg'}}$ are the intensity of the incoherent background for sample A and B, respectively. *P* is the intensity of the form factor at the peak position, and *S* is the value of the inter-particle structure factor at the peak position too. As the peak at 30 ˚C slightly shifts to high-q position compared to that at 20 ˚C and the packing of particles is different in the liquid state from that in the gel state, both the form factor and structure factor have different values at 20 ˚C and 30 ˚C. Taking the ratio of sample A and B, shown in Eq-3, removes the change of both the form factor and the structure factor due to slight change of the q-position of the scattering peak at those two temperatures. $\Delta\rho$ is the SLD difference between silica particles and its surrounding solvent in each temperature. $\varphi_{\mathrm{Lutidine,t}}$ and $\varphi_{\mathrm{matchedwater,t}}$ are the volume fractions of lutidine and water in the total sample volume, respectively. $\varphi_{\mathrm{Lutidine,d}}$ and $\varphi_{\mathrm{matchedwater,d}}$ are the volume fraction of lutidine and water in the particle domain, respectively. The intensity of the peaks is known from experiment and SLD can be calculated based on chemical formula and density. It is determined that the volume fraction of lutidine and water is 10.4 % and 89.6 % in the particle domain. It is worth mentioning that the SLD of the contrast matched water is experimentally using SANS by varying the SLD of water.

Supplementary Note 5: USANS patterns of a sample to demonstrate the thermal reproducibility, the long-time stability, and domain size tunability

Supplementary Fig. 3(a) shows two USANS patterns at 30 ˚C measured after cycling the temperature through 4 ˚C. The USANS patterns are completely identical, indicating that the domain structures are precisely reproducible when quenching rate is the same.

Supplementary Fig. 3(b) shows the USANS patterns of a sample in a gel state (at a fixed temperature of 30 ˚C) as a function of time. The USANS data do not show any change for a sample at a constant temperature for at least 20 hours, demonstrating that the domain structure is stable without aging effect in the time scale relevant for all the experiments conducted in this work.

Overall, SeedGel is thermally reversible with great structural reproducibility.

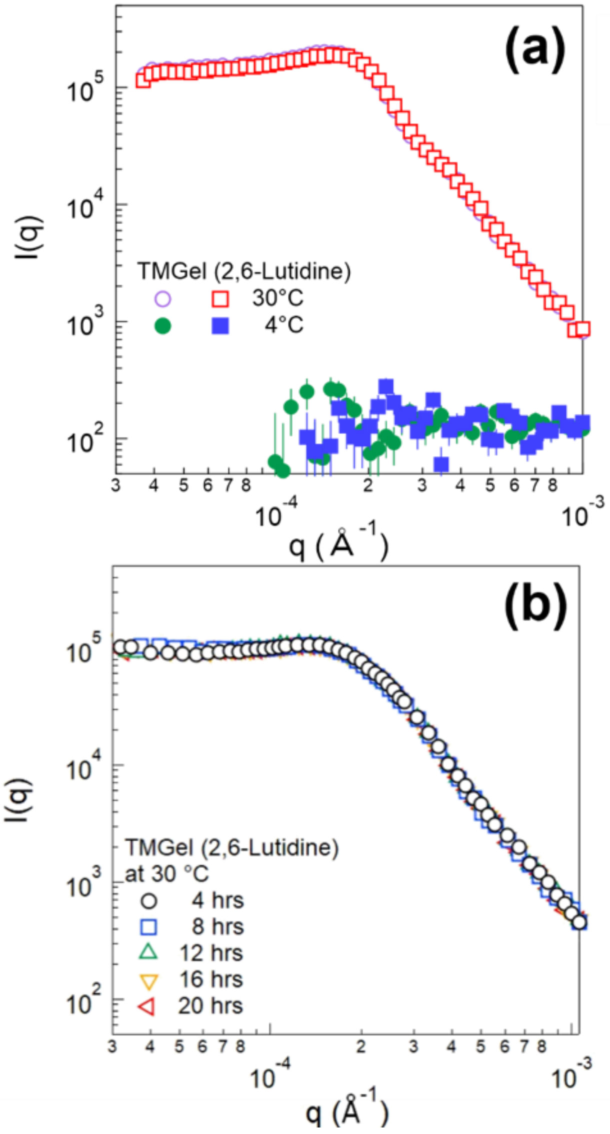

Supplementary Fig. 3. USANS profiles of SeedGel formed using 27 nm particles with (a) temperature cycling between 4 ˚C and 30 ˚C. (b) Stability test at 30 ˚C over a period of 20 hrs. The error bars in the figure represent one standard deviation.

Supplementary Note 6: Physics mechanisms of SeedGel formation

Supplementary Fig. 4 in the Supporting Information shows our proposed mechanism for the SeedGel formation. The local solvent phase separation initiates the attraction to drive the cluster formation that leads to the percolation of the particle domain (Supplementary Fig. 4(b) and (c)).

And, finally, the formation of the particle domain results in small sized pores (interstitial space between packed particles) that drive lutidine out of the particle domain. (Supplementary Fig. 4(d)) Thus, macroscopically, the water rich solvent region overlaps with the particle domain.

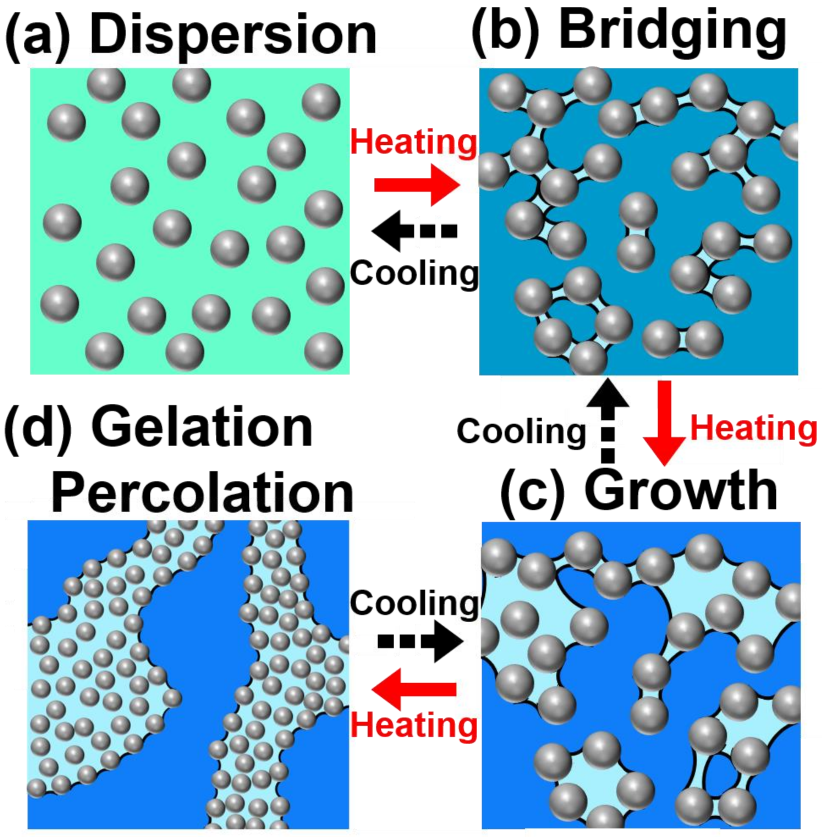

Supplementary Fig. 4 Schematic of SeedGel formation during solvent segregation upon heating. (a) At low temperatures, particles stay fully dispersed in a well-miscible binary solvent mixture. (b) and (c) Heating up the temperature causes local phase separation in between particles that attract them together due to capillary condensation. They form random sized aggregates that is visible by SANS. (d) Above the transition temperature, SeedGel is formed with particle domains percolating over the total sample volume. The whole process is completely reversible by cooling down the system.

Supplementary Note 7: Adjustable optical property of the SeedGel

As shown in Figure 2 (e) in the main text, the domain size remains unchanged at temperature range between 27 ˚C and 30 ˚C. Interestingly, the samples can be optically transparent, depending on the temperature (Supplementary Fig. 5). It is also noted that the tunable transparency can be achieved with different particle concentrations (Supplementary Fig. 6). This is due to the fact that the refractive index of the particle domain and solvent domain is close to each other as a result of relative partition of lutidine in both domains. The refractive indexes of lutidine (n=1.497) and silica (n=1.46) are similar to each other and are both larger than that of water (n=1.33). Higher temperature leads to a higher degree of phase separation between water and lutidine, resulting in higher lutidine ratio in the solvent domain. This increases the averaged refractive index of the solvent domain and reduces that in the particle domain. At a certain temperature, the two domains are optically indistinguishable, but a domain size of a few micrometers still persists (Figure 2e). The transparency to light provides opportunities for optically triggered reactions to occur within the bicontinuous channels that are difficult to be achieved by many other gel systems with micrometer sized domains.

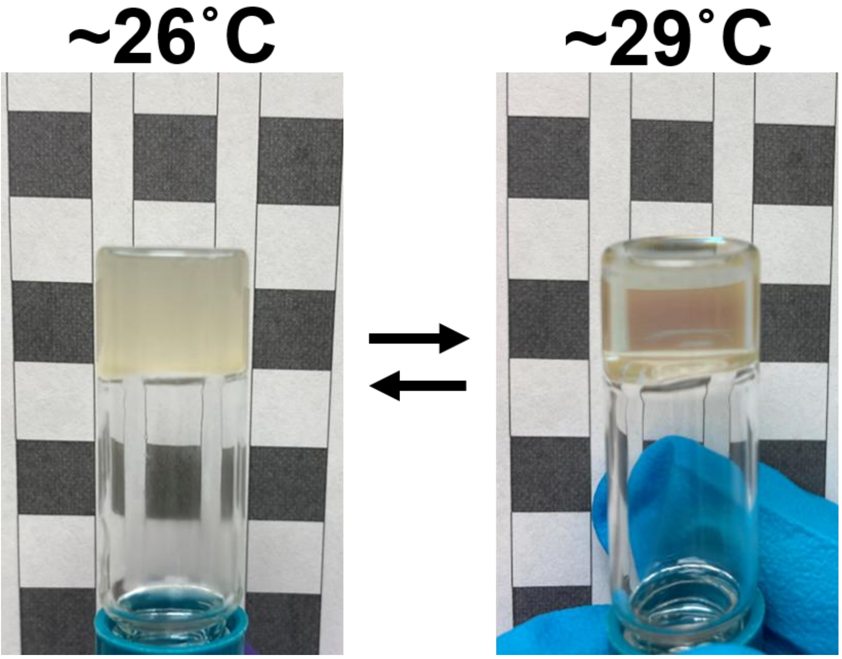

Supplementary Fig. 5. The transparency of SeedGel can be reversibly tuned by changing temperature. At certain temperatures, the gel becomes transparent even with micrometer sized domains. The particle volume fraction used in this sample is the same as that described in the main text, which is at 24.3 %.

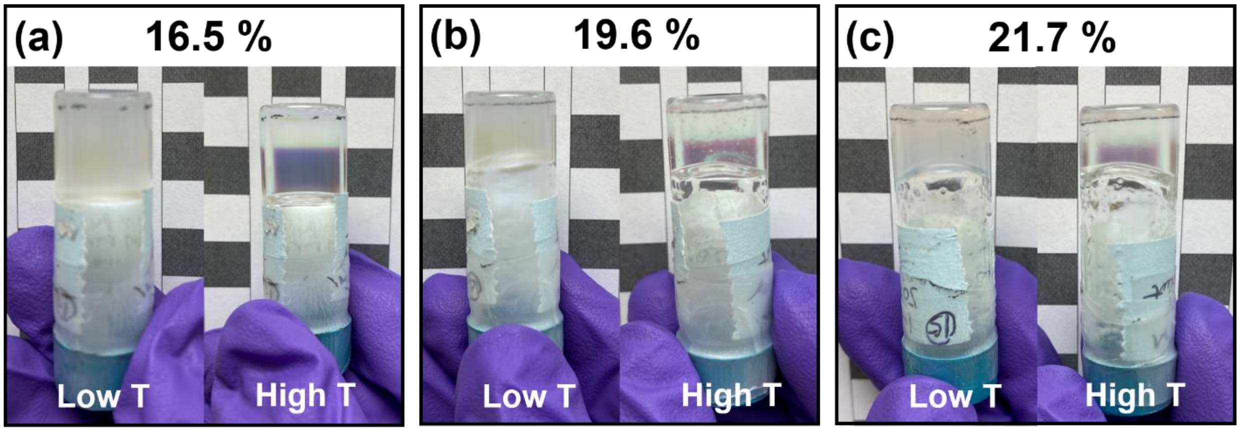

Supplementary Fig. 6. Transparent SeedGel can be obtained with particle volume fractions of (a) 16.5 %, (b) 19.6 % and (c) 21.7 %. The 2,6-lutidine weight fraction in solvent is fixed at 32.8 %. By changing the temperature, the transparency of the samples can be thermo-reversibly tuned. 'Low T' represents a low temperature around 26 ˚C and 'High T' means a high temperature near 29 ˚C.

Supplementary References:

1. Teubner, M. & Strey, R. Origin of the scattering peak in microemulsions. *J. Chem. Phys.* **87**, 3195–3200 (1987).
2. Schubert, K. -V., Strey, R., Kline, S. R. & Kaler, E. W. Small angle neutron scattering near Lifshitz lines: Transition from weakly structured mixtures to microemulsions. *J. Chem. Phys.* **101**, 5343–5355 (1994).
3. Endo, H. *et al.* Effect of amphiphilic block copolymers on the structure and phase behavior of oil–water-surfactant mixtures. *J. Chem. Phys.* **115**, 580–600 (2001).
4. Wang, Z., Guo, H., Liu, Y. & Wang, X. Investigating the effective interaction between silica colloidal particles near the critical point of a binary solvent by small angle neutron scattering. *J. Chem. Phys.* **149**, 084905 (2018).
5. Hayter, J. B. & Penfold, J. An analytic structure factor for macroion solutions. *Mol. Phys.* **42**, 109–118 (1981).
6. Hansen, J.-P. & Hayter, J. B. A rescaled MSA structure factor for dilute charged colloidal dispersions. *Mol. Phys.* **46**, 651–656 (1982).
7. Herzig, E. M., White, K. A., Schofield, A. B., Poon, W. C. K. & Clegg, P. S. Bicontinuous emulsions stabilized solely by colloidal particles. *Nat. Mater.* **6**, 966–971 (2007).
8. Bertrand, C. E., Godfrin, P. D. & Liu, Y. Direct observation of critical adsorption on colloidal particles. *J. Chem. Phys.* **143**, 084704 (2015).
9. Hertlein, C., Helden, L., Gambassi, A., Dietrich, S. & Bechinger, C. Direct measurement of critical Casimir forces. *Nature* **451**, 172–175 (2008).
10. Gambassi, A. *et al.* Critical Casimir effect in classical binary liquid mixtures. *Phys. Rev. E* **80**, 061143 (2009).
11. Burkhardt, T. W. & Eisenriegler, E. Casimir Interaction of Spheres in a Fluid at the Critical Point. *Phys. Rev. Lett.* **74**, 3189–3192 (1995).
12. Okamoto, R. & Onuki, A. Casimir amplitudes and capillary condensation of near-critical fluids between parallel plates: Renormalized local functional theory. *J. Chem. Phys.* **136**, 114704 (2012).
